# Development of a radiomics nomogram to predict the treatment resistance of Chinese MPO-AAV patients with lung involvement: a two-center study

**DOI:** 10.3389/fimmu.2023.1084299

**Published:** 2023-07-12

**Authors:** Juan Chen, Ting Meng, Jia Xu, Joshua D. Ooi, Peter J. Eggenhuizen, Wenguang Liu, Fang Li, Xueqin Wu, Jian Sun, Hao Zhang, Ya-Ou Zhou, Hui Luo, Xiangcheng Xiao, Yigang Pei, Wenzheng Li, Yong Zhong

**Affiliations:** ^1^ Department of Radiology, Xiangya Hospital, Central South University, Changsha, Hunan, China; ^2^ National Clinical Research Center for Geriatric Disorders, Xiangya Hospital, Central South University, Changsha, Hunan, China; ^3^ Department of Nephrology, Xiangya Hospital, Central South University, Changsha, Hunan, China; ^4^ Centre for Inflammatory Diseases, Monash University, Clayton, VIC, Australia; ^5^ Department of Nephrology, The third Xiangya Hospital, Central South University, Changsha, Hunan, China; ^6^ Department of Rheumatology and Immunology, Xiangya Hospital, Central South University, Changsha, China; ^7^ Key Laboratory of Biological Nanotechnology of National Health Commission, Xiangya Hospital, Central South University, Changsha, Hunan, China

**Keywords:** ANCA-associated vasculitis, myeloperoxidase, lung involvement, treatment resistance, radiomics nomogram

## Abstract

**Background:**

Previous studies from our group and other investigators have shown that lung involvement is one of the independent predictors for treatment resistance in patients with myeloperoxidase (MPO)–anti-neutrophil cytoplasmic antibody (ANCA)-associated vasculitis (MPO-AAV). However, it is unclear which image features of lung involvement can predict the therapeutic response in MPO-AAV patients, which is vital in decision-making for these patients. Our aim was to develop and validate a radiomics nomogram to predict treatment resistance of Chinese MPO-AAV patients based on low-dose multiple slices computed tomography (MSCT) of the involved lung with cohorts from two centers.

**Methods:**

A total of 151 MPO-AAV patients with lung involvement (MPO-AAV-LI) from two centers were enrolled. Two different models (Model 1: radiomics signature; Model 2: radiomics nomogram) were built based on the clinical and MSCT data to predict the treatment resistance of MPO-AAV with lung involvement in training and test cohorts. The performance of the models was assessed using the area under the curve (AUC). The better model was further validated. A nomogram was constructed and evaluated by DCA and calibration curves, which further tested in all enrolled data and compared with the other model.

**Results:**

Model 2 had a higher predicting ability than Model 1 both in training (AUC: 0.948 vs. 0.824; *p* = 0.039) and test cohorts (AUC: 0.913 vs. 0.898; *p* = 0.043). As a better model, Model 2 obtained an excellent predictive performance (AUC: 0.929; 95% CI: 0.827–1.000) in the validation cohort. The DCA curve demonstrated that Model 2 was clinically feasible. The calibration curves of Model 2 closely aligned with the true treatment resistance rate in the training (*p* = 0.28) and test sets (*p* = 0.70). In addition, the predictive performance of Model 2 (AUC: 0.929; 95% CI: 0.875–0.964) was superior to Model 1 (AUC: 0.862; 95% CI: 0.796–0.913) and serum creatinine (AUC: 0.867; 95% CI: 0.802–0.917) in all patients (all *p*< 0.05).

**Conclusion:**

The radiomics nomogram (Model 2) is a useful, non-invasive tool for predicting the treatment resistance of MPO-AAV patients with lung involvement, which might aid in individualizing treatment decisions.

## Introduction

Antineutrophil cytoplasmic antibody (ANCA)-associated vasculitis (AAV) is a serious autoimmune disease with multisystem involvement (1), which has a preference for affecting the kidney and lung (2) and is life threatening without treatment (3). AAV is commonly associated with the presence of ANCA against proteinase 3 (PR3) and myeloperoxidase (MPO) (1). MPO-AAV is the dominant form of AAV in China (4) with more than 90% MPO-AAV patients exhibiting kidney involvement and over 60% with pulmonary involvement. Initial renal function and pulmonary involvement are independent predictors of all-cause mortality in AAV (5, 6).

Currently, the outcome of AAV patients has dramatically improved with the introduction of steroids together with cyclophosphamide (CTX) or rituximab for induction therapy. However, 10%–30% of AAV patients remain resistant to this treatment (7). An increase in glucocorticoid dose and switching from cyclophosphamide to rituximab could be considered in AAV patients with treatment resistance (2). However, 96.9% of patients who were resistant to therapy progressed to end-stage renal disease (ESRD), and 50% of them died (8). Thus, the prediction of treatment resistance is crucial to personalize therapy for those AAV patients before therapy commencement, especially for MPO-AAV patients in China.

At present, there are some studies to forecast treatment resistance. Previous studies suggested that being female, black ethnicity, older age, and having elevated serum creatinine levels may be independent predictors of treatment resistance in patients with AAV (7–9). In addition, our previous study has shown that lung involvement is one of the independent predictors of treatment resistance in Chinese patients with MPO-AAV (8). Lung involvement in MPO-AAV was considered likely in the presence of hemoptysis, pulmonary hemorrhage, respiratory failure, and radiographic proof of infiltrates, nodules, or cavities without evidence of infection (10). However, it remains unclear which specific image features of lung involvement can predict a response to treatment in MPO-AAV patients, which is vital in decision-making for these patients.

Radiomics is a newly emerging form of imaging analysis that automatically extracts potentially unrecognizable information from medical images (11). Some reports suggest that radiomics could predict the therapeutic response and prognosis of interstitial lung disease (12, 13). Feng et al. (12) found that radiomics had a good predictive performance for interstitial lung disease treated by glucocorticoids. Yang et al. (13) proposed that radiomics could predict the response of antifibrotic treatment to idiopathic pulmonary fibrosis. These studies suggest that radiomics might be an effective tool to potentially predict the treatment resistance for MPO-AAV patients.

However, to the best of our knowledge, the use of radiomics analysis to forecast the treatment resistance for MPO-AAV patients with lung involvement has not been reported. Therefore, in this retrospective study, our aim is to develop a radiomics nomogram to predict the treatment resistance of MPO-AAV patients with lung involvement in a two-center study of Chinese patients.

## Methods

### Patient enrollments

This retrospective study was conducted by the Hunan Vasculitis Study Group (HuVas) in China. The study protocol followed the provisions of the Declaration of Helsinki and was approved by the Ethics Committee of each participating institution. Informed consent was obtained from all individual participants or their legally acceptable representatives.

**Figure 1:**
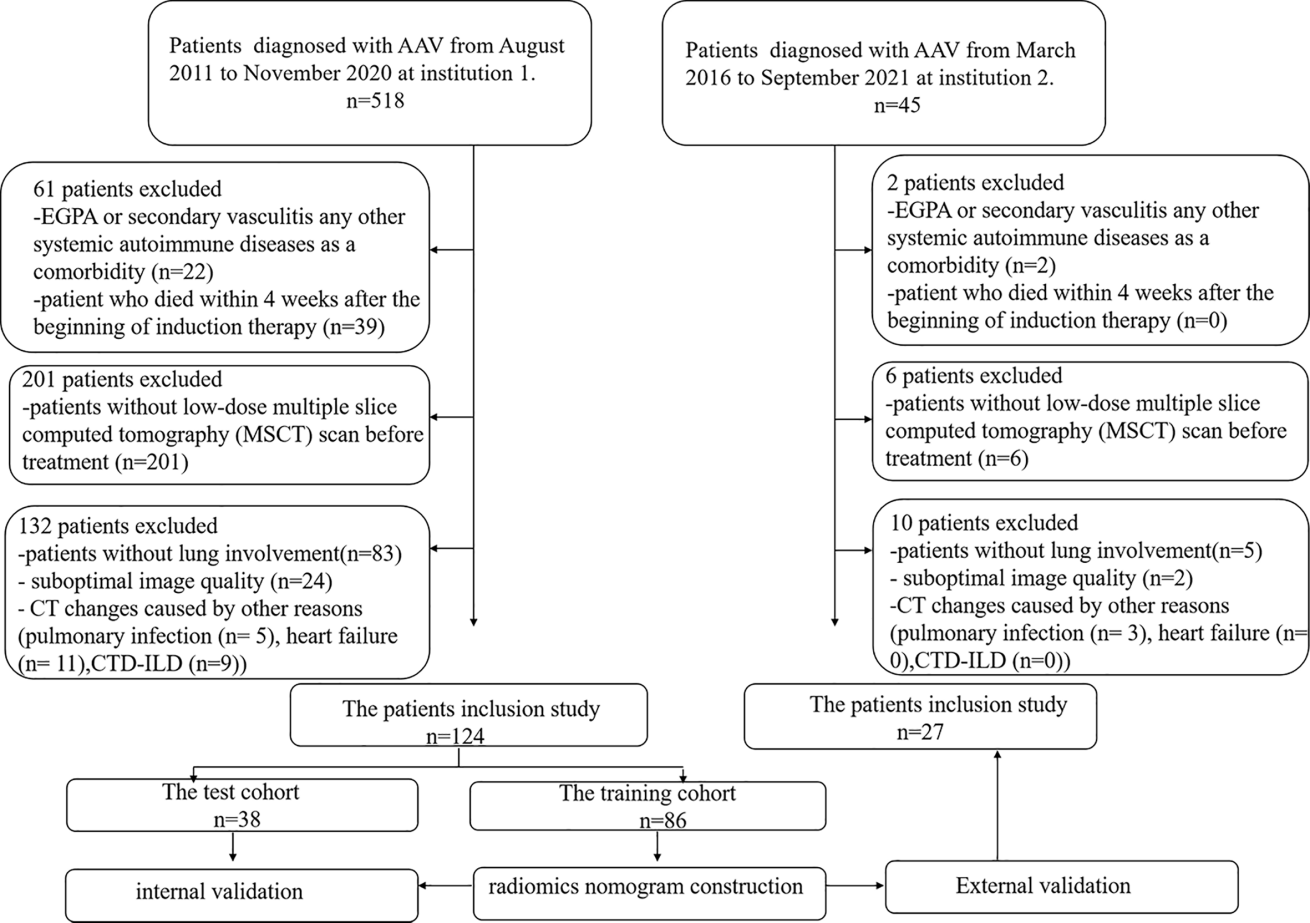
Workflow of patient enrollment.

We searched the database of two institutions to retrieve the radiological and clinical data of patients with confirmed MPO-AAV between August 2011 and September 2021. Consecutive patients with confirmed MPO-AAV were identified and enrolled as the following inclusion criteria: (1) a positive test for MPO-ANCA with the criteria of Chapel Hill Consensus Conferences Nomenclature of Vasculitis proposed in 2012 (14); (2) detailed clinical data; (3) multiple slices computed tomography (MSCT) imaging performed within 1 month before treatment; (4) sufficient image quality to allow accurate interpretation of radiological features; and (5) lung involvement on MSCT. Exclusion criteria were as follows: (1) eosinophilic granulomatosis with polyangiitis (EGPA), secondary vasculitis, or any other systemic autoimmune disease; (2) patients who died within 4 weeks after the beginning of induction therapy; (3) patients without a low-dose MSCT scan before treatment; (4) suboptimal image quality making the evaluation of imaging characteristics difficult; and (5) CT changes caused by other reason [such as pulmonary infection [clinical signs including fever, cough, and purulent secretions, with the presence of interstitial infiltrates, masses, cavitations, and abscesses in CT (15)], heart failure [cardiomegaly, ground glass opacification, different stages of pulmonary edema, pleural effusion, and increased peripheral vascular diameter in CT (16)], and connective tissue disease associated interstitial pneumonia (CTD-ILD)]. A total of 151 patients out of the 563 subjects were enrolled. The development cohort consisted of 124 patients who were diagnosed at institution 1 (The Xiangya Hospital of Central South University, Changsha, China). The validation cohort consisted of 27 patients who were diagnosed and treated at institution 2 (The Third Xiangya Hospital of Central South University, Changsha, China). Patient enrollment details are listed in **Figure 1**.

### MPO-AAV and therapy resistance

Birmingham Vasculitis Activity Score (BVAS) was used to measure disease activity (17). Organ system involvement was considered only if the manifestations were due to AAV (17). Particularly, lung involvement was considered likely in the presence of hemoptysis, pulmonary hemorrhage, respiratory failure, or radiographic proof of infiltrates, nodules, or cavities without evidence of infection (9, 18, 19). The estimated glomerular filtration rate (eGFR) was determined as described previously (20).

Serum ANCA was detected by both antigen-specific ELISA (Inova Diagnostics, San Diego, USA) and indirect immunofluorescence (IIF) (Euroimmun, Lübeck, Germany).

Treatment resistance was defined as unchanged or increased disease activity in patients with acute AAV after 4 weeks of treatment with standard therapy or a reduction of <50% in the disease activity score after 6 weeks of treatment or chronic, persistent disease defined as the presence of at least one major item or three minor items on the BVAS list after 12 weeks of treatment (21).

### Treatment process and therapy resistance

MPO-AAV patients received therapy, as previously described (22). In brief, oral glucocorticoids (prednisolone, starting at a dosage of 1 mg/kg daily for 4–6 weeks, tapered over 3–6 months to 12.5–15 mg/day) and cyclophosphamide (CTX) were administered intravenously at 0.5–0.75 g/m^2^ every month. For those over 65 years old or those with severe renal insufficiency, a 25% dose reduction of CTX was used, and CTX was temporarily stopped for those who developed leukocytopenia or thrombocytopenia. Some patients with rapidly progressive glomerulonephritis or pulmonary hemorrhage received an intravenous methylprednisolone pulse before the standard induction therapy and/or plasma exchange. Patients were followed up via phone and medical records to determine their status.

### The collection of clinical data

All patient demographic data and laboratory parameters were collected retrospectively from the electronic medical record system, including age (years), gender, white blood cells (10^9^/L), hemoglobin (g/L), platelet (10^9^/L), serum albumin (g/L), serum globulin (g/L), serum creatinine (μmol/L), erythrocyte sedimentation rate (ESR) (mm/h), C-reactive protein (CRP) (mg/L), C3 (mg/L), C4 (mg/L), IgA (mg/L), IgG (g/L), IgM (mg/L), alanine aminotransferase (ALT) (U/L), aspartate aminotransferase (AST) (U/L), total bilirubin (TBIL) (μmol/L), direct bilirubin (DBIL) (μmol/L), blood urea nitrogen (BUN) (mmol/L), neutrophil (10^9^/L), lymphocyte (10^9^/L), and eosinophil (10^9^/L) (**Table 1**). These laboratory data were obtained within 4 weeks before treatment.

**Table 1:** The clinical characteristics between treatment-resistant and treatment-responsive group.

	Treatment-resistant (n=55)	Treatment-responsive (n=96)	*p*
Age (years)	61 ± 12	61 ± 11	0.771
White blood cells (10^9^/L)	9.02 ± 4.30	10.26 ± 4.16	0.083
Hemoglobin (g/L)	77.36 ± 18.30	86.60 ± 19.81	0.005
Platelet (10^9^/L)	238.42 ± 101.08	298.19 ± 109.50	0.001
Serum albumin (g/L)	32.10(28.30–37.30)	31.45(26.75–36.33)	0.574
Serum globulin (g/L)	30.67 ± 7.77	33.07 ± 8.13	0.078
Serum creatinine (μmol/L)	643.28 ± 295.19	258.71 ± 242.61	<0.001
ESR (mm/h)	66.73 ± 36.59	71.36 ± 40.30	0.484
CRP (mg/L)	18.40(7.25–68.20)	38.14(5.77–95.85)	0.188
C3 (mg/L)	693.40 ± 261.76	802.48 ± 342.59	0.030
C4 (mg/L)	214.33 ± 105.50	218.97 ± 105.86	0.796
IgA (mg/L)	2413.93 ± 1435.34	2635.28 ± 1572.43	0.392
IgG (g/L)	14.04 ± 5.96	15.61 ± 5.54	0.104
IgM (mg/L)	860.00 ± 456.19	1054.42 ± 630.60	0.047
ALT (U/L)	9.90(5.40–14.30)	14.90(8.90–24.13)	0.001
AST (U/L)	16.00(12.60–25.90)	18.60(14.90–31.88)	0.046
TBIL (μmol/L)	5.30(4.20–7.00)	6.60(4.70–8.70)	0.024
DBIL (μmol/L)	2.50(1.90–3.80)	2.90(1.90–4.08)	0.393
BUN (mmol/L)	21.04 ± 10.54	11.62 ± 8.53	0.001
Neutrophil (10^9^/L)	7.10 ± 3.92	8.10 ± 4.85	0.197
Lymphocyte (10^9^/L)	1.00(0.60–1.40)	1.10(0.70–1.60)	0.519
Eosinophil (10^9^/L)	0.12(0.03–0.20)	0.10(0.00–0.30)	0.451
Total Prednisolone, g Median(Q1,Q3)	4.69(3.60,5.00)	4.96(4.95,6.30)	<0.001
Total CTX, g Median(Q1,Q3)	2.40(1.80,3.60)	4.80(3.60,4.80)	<0.001
MP, n (%)	14 (25.5%)	12 (12.5%)	0.043
PE, n (%)	25 (45.5%)	22 (22.9%)	0.004

ESR, erythrocyte sedimentation rate; CRP, C-reactive protein; ALT, alanine aminotransferase; AST, aspartate aminotransferase; TBIL, total bilirubin; DBIL, direct bilirubin; BUN, blood urea nitrogen; CTX, cyclophosphamide; MP, methylprednisone pulse; PE, plasma exchange.

### Evaluation of lung involvement on MSCT

**Figure 2:**
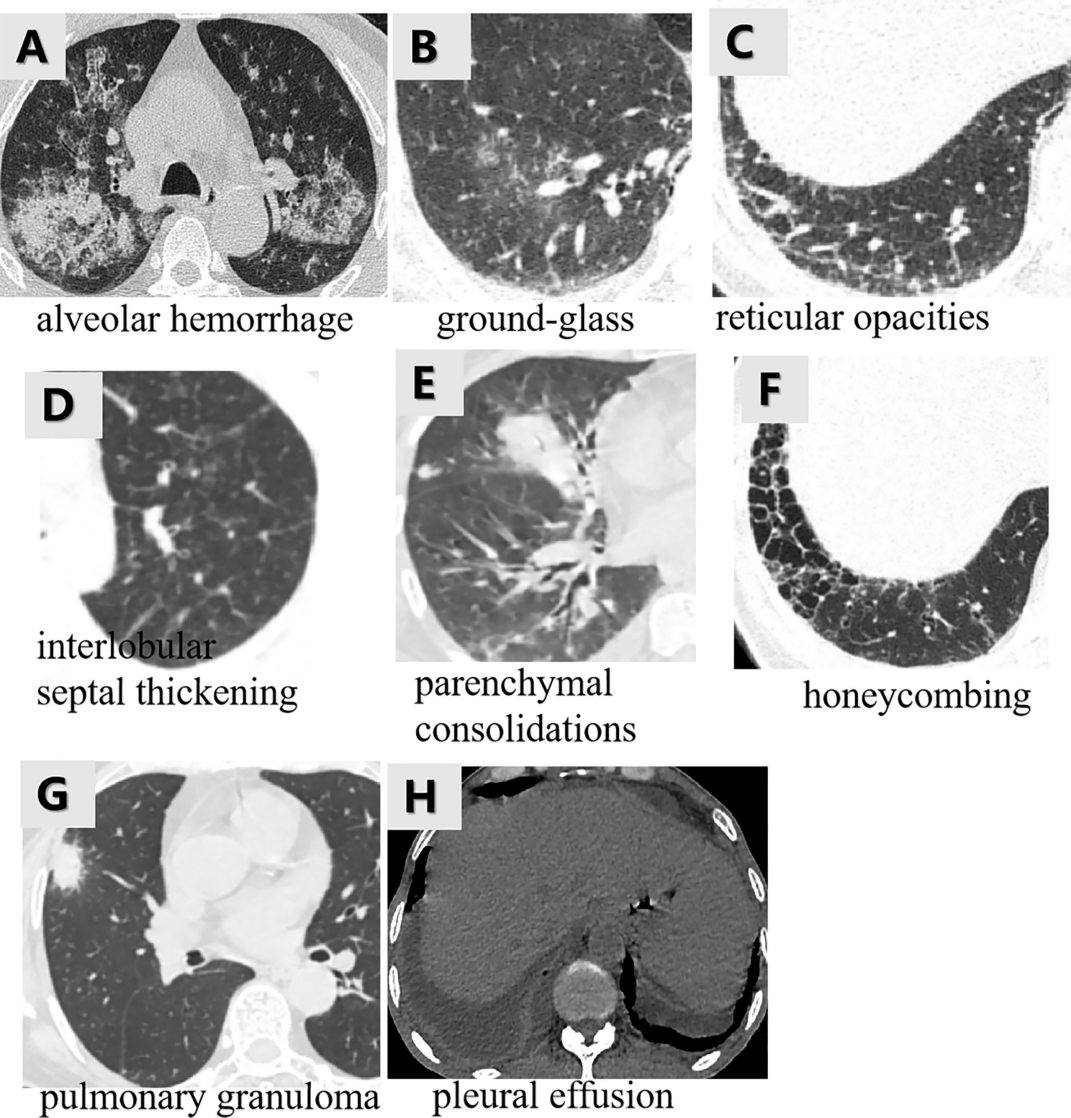
The various imaging features of MPO-AAV patients with lung involvement in MSCT. Alveolar hemorrhage was the appearance of diffuse pulmonary infiltrates with bilateral opacities, ground-glass, and crazy-paving pattern **(A)**; interstitial lung disease including the five imaging features **(B–F)**; pulmonary granuloma surrounded with halo sign, manifested as a nodule, mass, or cavity and ranged from a few millimeters to more than 10 cm in diameter **(G)**; bilateral pleural effusion **(H)**.

The MSCT images with lung involvement were collected for those MPO-AAV patients from the picture archiving and communication system (PACS). The image acquisition parameters are shown in detail in **Appendix E1** and **Supplementary Table S1**. Two radiologists, reader 1 and reader 2, with 5 and 15 years of thoracic radiology experience, respectively, reviewed all MSCT images and assigned the following qualitative features for each patient: (a) alveolar hemorrhage (AH), the appearance of diffuse pulmonary infiltrates with bilateral opacities, ground-glass, and crazy-paving pattern (2) (**Figure 2A**); (b) interstitial lung diseases (ILD) including ground glass (**Figure 2B**), reticular opacities (**Figure 2C**), interlobular septal thickening (**Figure 2D**), parenchymal consolidations (**Figure 2E**) and honeycombing (**Figure 2F**) (23); (c) pulmonary granuloma, manifested as a nodule, mass, or cavity and ranged from a few millimeters to more than 10 cm in diameter (24) (**Figure 2G**); and (d) pleural effusion (**Figure 2H**). The two radiologists were blinded to the clinical and pathological information for evaluating lung involvement of MPO-AAV patients. Any disagreement was resolved through consultation.

### Independent predictors acquisition

Univariate analysis was used to compare the differences in clinical factors and qualitative MSCT features between treatment-resistant and treatment-responsive groups. A p-value< 0.05 indicates a significant difference. The significant factors were entered into a multivariate analysis to select the independent predictors. Odds ratio (OR) and 95% confidence intervals (CIs) for each independent predictor were computed.

### Building of Model 1 (radiomics signature)

#### Region of interest segmentation

3D segmentation of the primary lung lesions was performed by two readers (reader 1 and reader 2, with 5 and 15 years of experience in thoracic imaging, respectively) for the regions of interest (ROIs) that were manually or semi-automatically delineated on the MSCT images based on the threshold method and edge-based method by using 3D-slicer software (version 4.8.1; http://www.slicer.org) (2).

For those lesions with unclear borders, such as alveolar hemorrhage and interstitial lung disease, the threshold method was applied to sketch them with the Hounsfield unit (HU) values (−700 to −150 HU). Manual corrections were carried out when the automatically registered borders did not correspond to the actual lesion’s margin (25, 26). The criteria of manual modification were as follows: 1) for those lesions that were not covered completely in ROI, including patch and nodules, manual delineation was applied; 2) for those lung lesions that were not included in the ROI automatically, manual delineation was applied; 3) the main and leaf bronchi were not contained in the ROI, such as when the segmental and inferior bronchi were connected to pixels that were distinguishable by the naked eye, meaning that they would not be sketched into the ROI. The small scattered bronchus of the lungs was contained in the ROI; 4) the hilar vessels were carefully excluded (27).

For the distinct boundary-pulmonary granuloma, ROI was manually contoured along the boundary of the lesion in every slice (28). The vascular or bronchial structure were included within the segmentation when they were surrounded by the lesion and excluded when they were close to the lesion’s edge (29). The lesion in the last slices was not included to avoid volume averaging with adjacent structures. Initially, both readers independently segmented 30 randomly selected patients (including 18 treatment-resistant and 12 treatment-responsive subjects). After that, a reader (*BLINDED*) repeated the same segmentation a week later to obtain intra- and inter-rater intra-class correlation coefficients (ICC) as described by (30). Texture features with ICC > 0.75 were considered to have a good agreement. Reader 1 continued with the remaining image segmentation.

#### Radiomic feature extraction

The ROIs and original images were transferred into the radiomics platform AK software 3.3.0 (Analysis kit, GE Healthcare, China) for image preprocessing. All MSCT images and segmented ROIs were resampled to 1.0×1.0×1.0 mm^3^ voxel size to standardize the voxel spacing. A Gaussian filter of 0.5 mm bandwidth was used to filter noise from the images. Radiomics features including shape (n=14), first-order (n=18), second-order [glcm(n=24), glrlm (n=16), glszm (n=16), ngtdm (n=5), gldm (n=14)] and higher-order (wavelet transform) (n=744) features, which were confirmed to reflect heterogeneity of lesions and potentially reflected changes in image structure, were extracted from MSCT images (31). Wavelet transform (sigma = 2.0, 3.0) filters (n=8) were selected to transform features. A total of 851 features were extracted for each patient (**Supplementary Table S2**).

To select robust radiomics features, the features with outliers (under the first quartile or above the third quartile of the feature distribution) and missing values were replaced by the feature’s median value in the dataset. Finally, all features were standardized using zero-mean normalization to remove pixels that fall outside a specified range of gray levels.

### Construction of Model 1

The development cohort (patients from institution 1) was randomly split into training and test cohorts at a ratio of 7:3. First, the radiomics features with ICC > 0.75 from the MSCT images in the training cohort were selected to train the predictive model. Subsequently, the variance threshold method was used to remove variance with a value<0.8. Thereafter, the features were entered into the multivariate logistic regression to select the robust features. The radiomics score (Rad-score) for each patient was calculated based on the robust features with a calculation formula (**Appendix E2**). Model 1 (radiomics signature) was thus built based on Rad-score and further tested in the test cohort. Odds ratio (OR) and 95% confidence intervals (CIs) for the Rad-score were evaluated.

### Construction of Model 2

**Figure 3:**
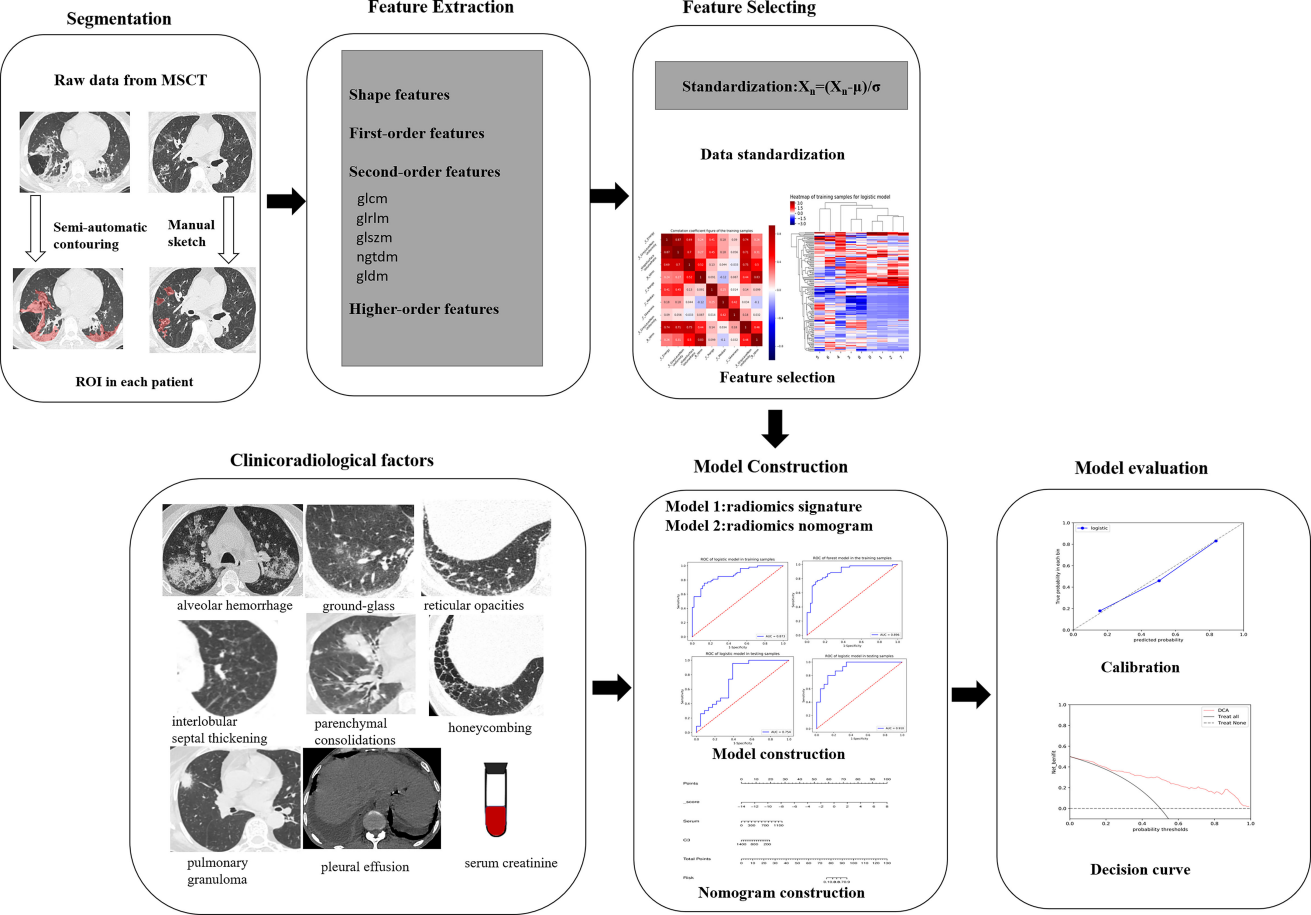
Workflow of the radiomics signature building and model construction.

A combined radiomics model (Model 2) was built by the independent clinical predictors combined with the Rad-score, whose predictive performance was analyzed in the training and test cohorts (institution 1). A better model was chosen with the maximum area under the curve (AUC) between Model 1 and Model 2. The workflow of model construction is shown in **Figure 3**.

### The clinical utility of the better model

The better model was further validated in the validation cohort (institution 2). A decision curve analysis (DCA) was used to estimate the clinical utility of the better models by calculating the net benefits for a range of threshold probabilities (percentage risk threshold of detecting the subtype). For each decision curve, the net clinical benefit was computed using the formula (32):


$$\text{Net benefit}=\;\frac{True\;positives}{N}-\frac{False\;positives}{N}\times \frac{p_{t}}{1-p_{t}}$$

where *p_t_* is the threshold probability for detecting a positive patient.

The decision curve plots net clinical benefit (y-axis) against threshold probability (x-axis). The clinical utility of the curve is indicated by the highest net clinical benefit at the lowest threshold probability.

A nomogram for the better clinically applicable model was also constructed based on the AUC performance and clinical utility at the lower threshold probability. The process of graphical presentation of the nomogram is described in **Appendix E3**.

The predictive performance of the better model was also compared with the other model and the previous independent predictors in all enrolled patients (institutions 1 and 2), respectively.

### Statistical analysis

All data were analyzed using the statistical software SPSS (version 22, IBM SPSS Inc., Chicago) and R statistical software (Version 3.4.1, http://www.Rproject.org). Baseline characteristics were presented as means and standard deviations (SDs) or median with interquartile range for continuous variables and percentages for categorical variables. It was considered statistically significant when the *p*-value was < 0.05. Univariate analyses were used to compare differences between the treatment-resistant and treatment-responsive groups regarding clinical–radiological characteristics using chi-squared or Fisher’s exact tests for categorical variables and Mann–Whitney U test for continuous variables. The diagnostic performance of the models in differentiating treatment-resistant patients from treatment-responsive patients was assessed by the AUC (with 95% CI), accuracy (ACC), sensitivity (SEN), and specificity (SPE), in training, test, and validation cohorts. A model is considered to have excellent, good, or poor performance when it has an AUC of 0.85–1.0, 0.7–0.85, or<0.7, respectively (33). The curves of the models were compared using the Delong test. Decision curve analysis (DCA) compared the net benefits under different threshold probabilities given by the better model. Calibration curves of the better model were drawn to evaluate the consistency between the predicted results and the real results.

## Results

### Basic clinical–radiological characteristics

A total of 151 patients (mean age 60 ± 11 years; 70 men and 81 women) were enrolled, including 55 patients (mean age 60 ± 12 years; 26 men and 29 women) with treatment resistance and 96 subjects (mean age 61 ± 11 years; 44 men and 52 women) showing a response to treatment. They were divided into a training cohort (86 patients of mean age 60 ± 13 years; 38 men and 48 women; 33 patients with treatment resistance and 53 subjects with treatment response), a test cohort (38 patients of mean age 61 ± 14 years; 21 men and 17 women; 15 patients with treatment resistance and 23 patients with treatment response), and validation cohort (27 patients of median age, 63 ± 8 years; 16 men and 11 women; 7 treatment-resistant and 20 treatment-responsive), respectively (**Figure 1**). For clinical data, the levels of serum platelets, creatinine, C3, IgM, ALT, AST, TBIL, and BUN were higher in the treatment resistance group than in the treatment response cohort (*p*<0.05) (**Table 1**). For example, the value of serum creatinine in the treatment resistance cohort was much higher compared to the treatment response group (*p*< 0.001). The total CTX dose was 2.40 g (1.80,3.60) in treatment-resistant group and 4.80 g (3.60,4.80) in treatment-responsive group. The cumulative dose of CTX were significant lower in treatment-resistant group than in treatment-responsive group (*p<*0.001) (**Table 1**). Since 21 of the 55 patients in the treatment-resistant group were on dialysis at onset and remain dialysis-dependent after 3 months of induction therapy. According to KDIGO Guideline (34, 35), the immunosuppressive medication therapy was discontinued in these dialysis-dependent patients. For radiological features, interlobular septal thickening, honeycombing, and pleural effusion were significantly different in the treatment-resistant group compared with the treatment-responsive set (*p*<0.05) (**Table 2**). Finally, the serum creatinine (OR: 1.004; 95% CI: 1.002–1.006, *p* < 0.001) remained as an independent predictor of treatment resistance with multivariate analysis (**Table 3**).

**Table 2:** The imaging features of patients between treatment-resistant and treatment-responsive group.

	Treatment-resistant (n=55) n (%)	Treatment-responsive (n=96) n (%)	*p*-value
Alveolar hemorrhage			0.098
Y	14(25.5)	14(14.6)	
N	41(74.5)	82(85.4)	
Interstitial pneumonia			0.001
Y	7(12.7)	37(38.5)	
N	48(87.3)	59(61.5)	
Ground-glass			0.077
Y	34(61.8)	45(46.9)	
N	21(38.2)	51(53.1)	
Reticular opacities			0.050
Y	2(3.6)	13(13.5)	
N	53(96.4)	83(86.5)	
Interlobular septal thickening			0.034
Y	14(25.5)	41(42.7)	
N	41(74.5)	55(57.3)	
Parenchymal consolidations			0.176
Y	10(18.2)	10(10.4)	
N	45(81.8)	86(89.6)	
Honeycombing			0.012
Y	5(9.1)	25(26.0)	
N	50(90.9)	71(74.0)	
Pulmonary granuloma			0.120
Y	3(5.5)	13(13.5)	
N	52(94.5)	83(86.5)	
Pleural effusion			0.002
Y	19(34.5)	13(13.5)	
N	36(65.5)	83(86.5)	

**Table 3:** Multivariable predictors of treatment resistance.

Predictors	*p*	OR (95%CI)
Serum creatinine	0.000	1.004(1.002–1.006)
Rad-score	0.000	2.995(1.877–4.780)

OR, odds ratio; CI, confidence interval.

### Performance of Model 1 (radiomics signature)

**Figure 4:**
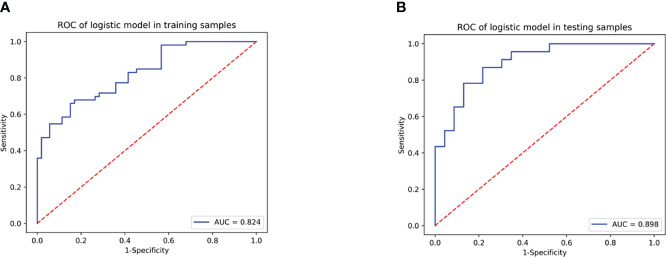
The predicting efficiency of Model 1 in the training cohort **(A)** and test cohort **(B)**.

A total of 355 features with ICC > 0.75 were selected from 851 texture features. Features with variance > 0.8 were screened for further analysis. After multivariate logistic analysis, nine optimal features (original_shapeSurfaceVolumeRatio, wavelet-LLH_firstorder_Energy, wavelet-HLH_firstorder_Range, wavelet-HHH_firstorder_Median, wavelet-HLH_firstorder_Skewness,wavelet-HHL _glszm_GrayLevelNonUniformity, wavelet-LHL_glcm_Idmn, wavelet-LLL_glszm_GrayLevelNonUniformity, and wavelet-HLL_glcm_Idmn) were shown to be distinctly associated with the treatment-resistant cohort (**Supplementary Table S3**). Rad-scores based on the above nine features are presented in (**Appendix E4**, **Supplementary Figure S1**) and used to build Model 1. Model 1 presented an excellent predictive performance in the training cohort (AUC: 0.824; 95% CI: 0.757–0.883; ACC: 0.717; SEN: 0.585; SPE: 0.849) and test cohort (AUC: 0.898; 95% CI: 0.816-0.962; ACC: 0.804; SEN: 0.826; SPE: 0.783) (**Table 4**, **Figure 4**). The OR value was 2.995, and 95% CI was 1.877–4.780 for the Rad-score with multivariate analysis (*p* < 0.01).

**Table 4:** ROC curve analysis of Models 1 and 2.

Training cohort					Test cohort				Validation cohort			
	AUC (95%CI)	SEN	SPE	ACC	AUC (95%CI)	SEN	SPE	ACC	AUC (95%CI)	SEN	SPE	ACC
Model 1	0.824 (0.757–0.883)	0.585	0.859	0.717	0.898 (0.816–0.962)	0.826	0.783	0.804	–	–	–	–
Model 2	0.948 (0.908–0.979)	0.939	0.792	0.849	0.913 (0.835–0.975)	0.867	0.783	0.816	0.929 (0.827–1.000)	0.714	0.950	0.889

ROC, receiver operating characteristic; CI, confidence interval; SEN, sensitivity; SPE, specificity; ACC, accuracy.

### Performance of Model 2 (radiomics nomogram)

**Figure 5:**
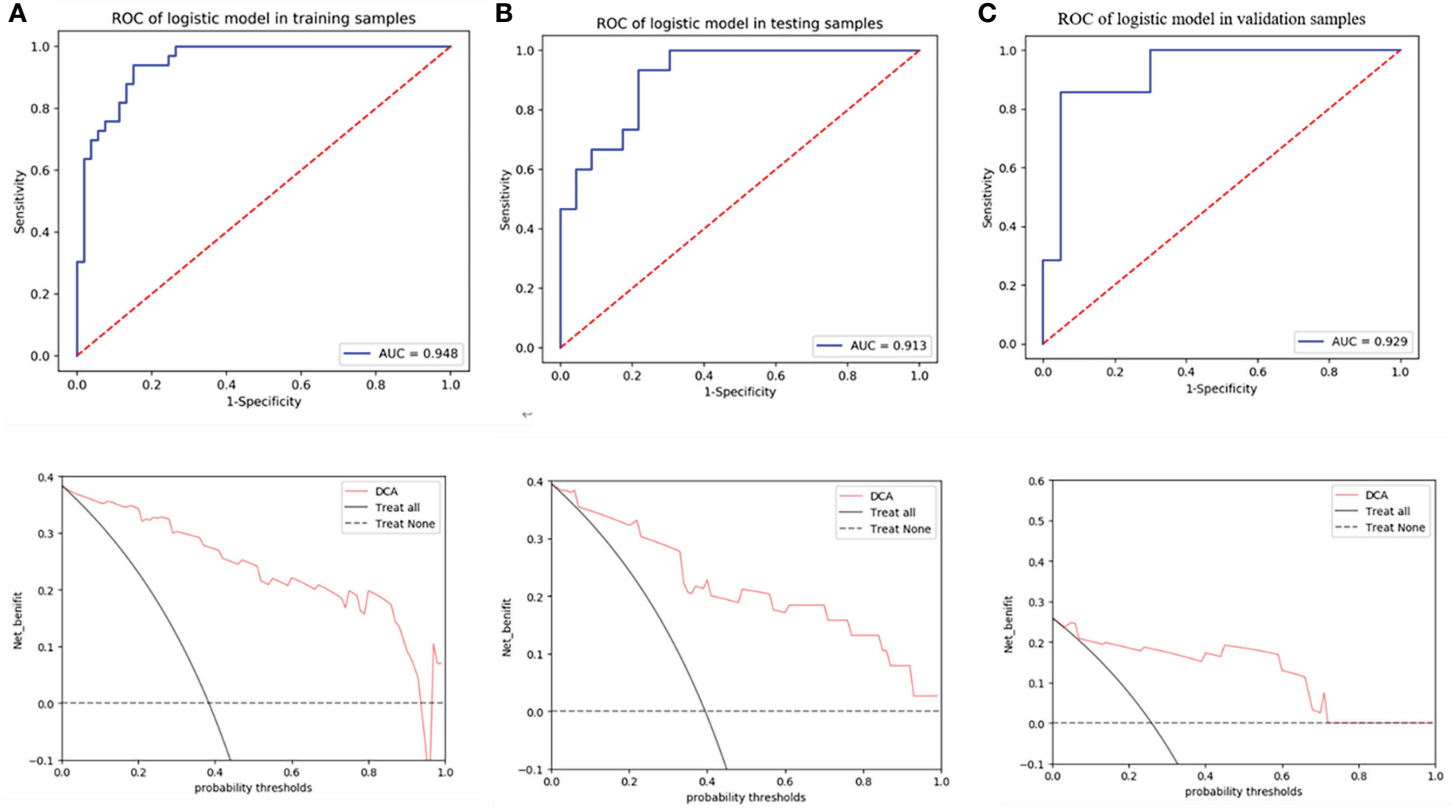
ROC curves of radiomics nomogram and decision curve analysis to detect the presence of treatment resistance in the training **(A)**, test **(B)**, and validation **(C)** cohorts, respectively.

**Figure 6:**
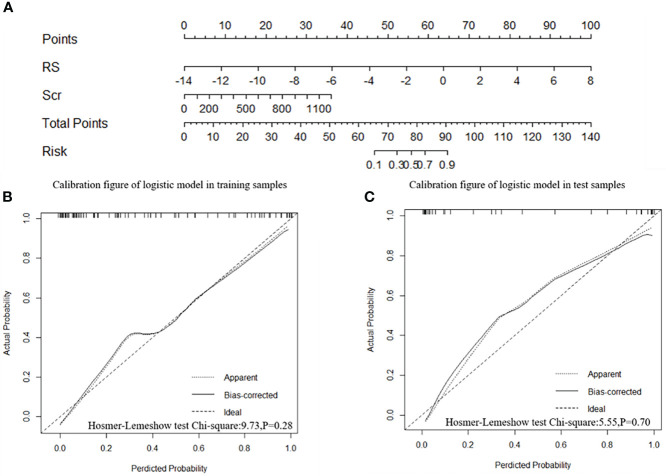
Radiomics nomogram and calibration curves. **(A)** The radiomics nomogram was developed in the training cohort with the Rad-score and serum creatinine. The calibration curve of the radiomics nomogram for treatment resistance in the training cohort **(B)** and test cohort **(C)**, respectively.

Model 2 showed an excellent predictive performance in the training (AUC: 0.948; 95% CI: 0.908–0.979; ACC: 0.849; SEN: 0.939; SPE: 0.792) and test cohort (AUC: 0.913; 95% CI: 0.835–0.975; ACC: 0.816; SEN: 0.867; SPE: 0.783), which was better than Model 1 in the training (*p* = 0.039) and validation set (*p* = 0.043), respectively (**Table 4**, **Figure 5**). Furthermore, Model 2 (better model) also obtained an outstanding predictive efficiency (AUC: 0.929; 95% CI: 0.827–1.000; ACC: 0.889; SEN: 0.714, SPE: 0.950) in the validation cohort (**Table 4**, **Figure 5**). The DCA curve of Model 2 showed that if the threshold probability of a patient was >5%, using Model 2 to predict treatment resistance of MPO-ANCA patients with lung involvement added more benefit than either the treat-none scheme or the treat-all-patients scheme in the three cohorts (**Figure 5**). A nomogram was constructed for Model 2, as it was easier to implement in routine clinical practice (**Figure 6A**). Its predicted probabilities closely aligned with the true treatment resistance rates in both training (*p* = 0.28) and test (*p* = 0.70) cohorts (**Figures 6B, C**).

**Figure 7:**
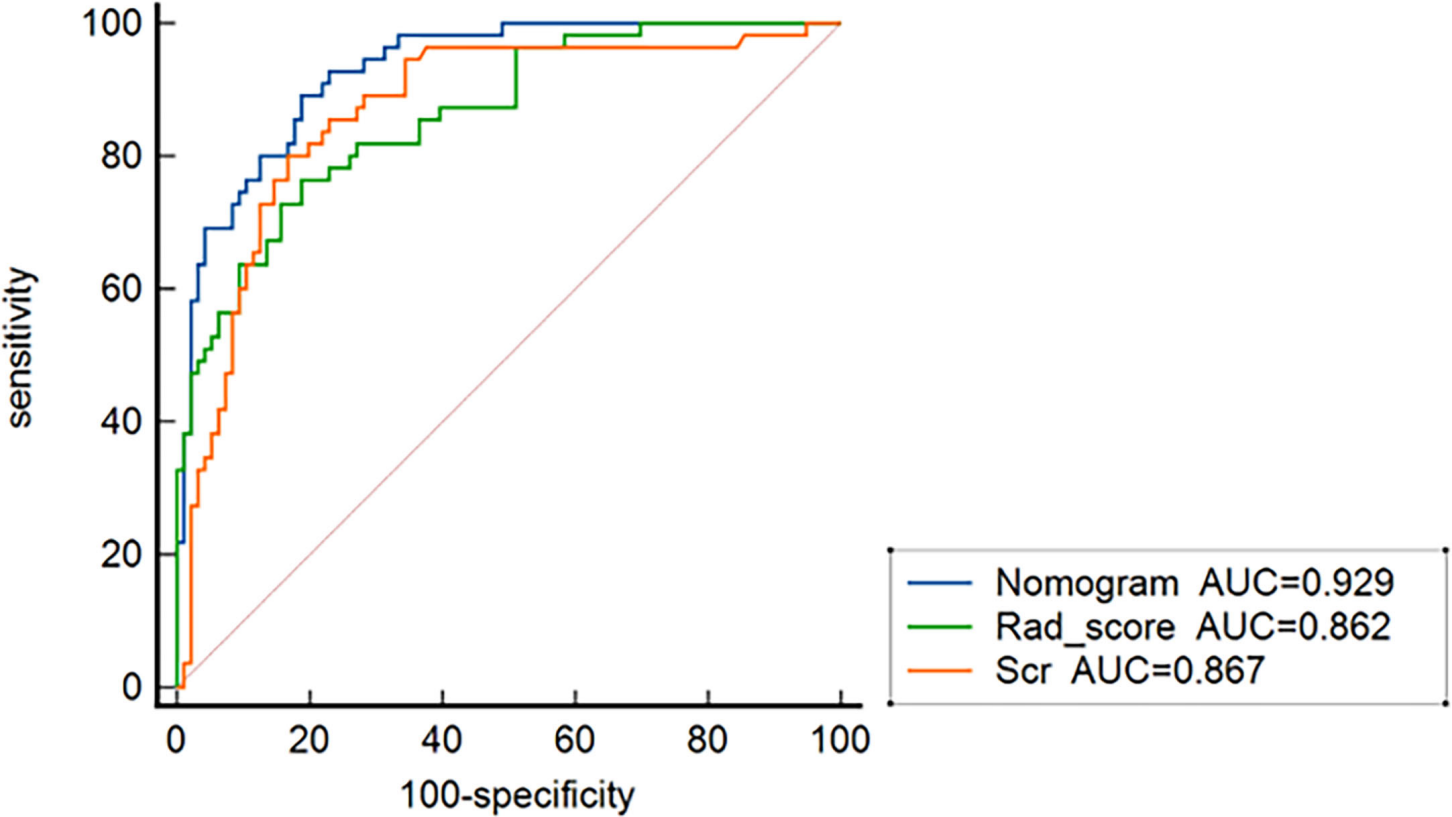
ROC curves for nomogram, the radiomics signature, and serum creatinine for predicting treatment resistance in all 151 patients.

In all patients, the predictive efficiency of Model 2 (AUC: 0.929; 95% CI: 0.875–0.964) was superior to that of Model 1 (AUC: 0.862; 95% CI: 0.796–0.913) (*p*<0.01) and serum creatinine (AUC: 0.867; 95%CI: 0.802–0.917) (*p* = 0.02), respectively (**Figure 7**).

## Discussion

At present, 10%–30% of AVV patients suffer from treatment resistance after 4 weeks of standard therapy (7, 36). Predicting treatment resistance is significant to monitor strategies and weigh up the relative benefits of different treatment strategies for MPO-AAV patients with lung involvement. Thus, we developed and validated various predictive models based on MSCT and clinical data to predict the treatment resistance for MPO-AAV patients with lung involvement before therapy.

In our study, the elevated serum creatinine level was the independent predictor of treatment resistance for MPO-AAV patients with lung involvement, which was in keeping with the findings of previous studies (7, 37). Li et al. (7) proposed that the poor response to treatment was associated with reduced renal function. The worse the renal function, the higher the level of serum creatinine, which causes a greater incidence of treatment resistance. Patients with a high level of serum creatinine had already sustained chronic, irreversible renal damage and interstitial scarring, which results in resistance to immunosuppressive therapy (9). Our results indicated that serum creatinine level can be a biomarker for predicting the treatment resistance of MPO-AAV patients with lung involvement.

In our study, we observed that interstitial lung disease (interlobular septal thickening, honeycombing) and pleural effusion performed a significant difference between the treatment-resistant group and the treatment-responsive set. The emergence of interstitial lung disease is a favorable factor for treatment response in our study. It is plausible that certain agents, such as rituximab, may have a beneficial effect as seen in CTD-ILD (38). It was observed that pleural effusion was more frequent in treatment-resistant patients, which might relate to MPO-ANCA activity (39). However, qualitative radiological features were not an independent predictor of treatment resistance for MPO-AAV patients with lung involvement, which was different to the findings that lung involvement is an independent predictor of treatment resistance in MPO-AAV patients (8). The most plausible explanation for this discrepancy might be that lung involvement includes various radiological features, including alveolar hemorrhage and interstitial lung disease. The lung involvement can be an independent factor of MPO-AAV patients, but their specific imaging features might not be. In addition, the enrollment of patients included some MPO-AAV patients without lung involvement in our previous report (8), which was different from the present study and led to selection bias.

Nine vital radiomics features (original_shapeSurfaceVolumeRatio, wavelet-LLH_firstorder_Energy, wavelet-HLH_firstorder_Range, wavelet-HHH_firstorder_Median, wavelet-HLH_firstorder_Skewness, wavelet-HHL _glszm_GrayLevelNonUniformity, wavelet-LHL_glcm_Idmn, wavelet-LLL_glszm_GrayLevelNonUniformity, and wavelet-HLL_glcm_Idmn) were associated with treatment resistance for those MPO-AAV patients with lung involvement, including one shape feature (shapeSurfaceVolumeRatio) (SVR), eight wavelet features {four features from first-order features (Energy, Range, Median, Skewness) and four features from textural features [Gray-Level Size Zone Matrix (GLSZM), Gray-Level Co-occurrence Matrix (GLCM)]}. A greater SVR indicates more spiculated and irregular lesions (40), which indicated a poor response to treatment. Energy refers to the magnitude of voxel values in the image, which could predict the earlier treatment response (41). In our study, it relates to the treatment resistance of patients with 12 weeks standard treatment. Lesion volume and maximum diameter had the highest predictive performance in response to treatment with Gefitinib for non-small-cell lung carcinoma patients (41), which indicated that the range and median of the lesion can predict treatment resistance for those MPO-AAV patients with lung involvement. Higher skewness occurs when a lesion contains regions of different intensities, implying greater lesion heterogeneity in the treatment-resistant group. Hötker et al. (42) found that the skewness could identify nephroblastoma patients at risk of poor response to treatment early. Our results implied that skewness could forecast the treatment resistance for the MPO-AAV patients with lung involvement early.

GLCM analyzed the spatial distribution of image texture features through different spatial positions and angles with 0°, 45°, 90°, and 135° as the angles generally used (43). The GLCM features can clearly predict the degree of lung injury (none/mild/severe) after stereotactic body radiotherapy at three various time points (3, 6, and 9 months) (44), which is in accordance with the finding that it can forecast the treatment resistance in our study. GLSZM quantifies gray-level zones in an image, and GLN (GrayLevelNonUniformity) from GLSZM measures the variability of gray-level intensity values in the image (45), which indicates the heterogeneity in the lung involvement lesion and reflects the resistance to treatment for those MPO-AAV patients.Model 2 has a higher predictive performance than Model 1 in both training and test cohorts. It is in keeping with several previous studies. Ligero et al. (46) reported that the radiomics clinical model improved the predictive performance to immune checkpoint inhibitors in advanced solid tumors compared with only radiomics model (AUC: 0.74 vs. 0.70; *p*< 0.001]. Another study found that the radiomics nomogram established by integrating the radiomics signature with clinical data outperformed the clinical nomogram alone in predicting induction chemotherapy response of nasopharyngeal carcinoma patients. (C-index in validation cohort: 0.863 vs. 0.549; *p*< 0.01) (47). This study has demonstrated the combined model (Model 2) benefit MPO-AAV patients with lung involvement by adding a prediction of treatment resistance.

As a better model, Model 2 was beneficial in detecting treatment resistance at a lower threshold probability of 5%, which is a lower threshold than the reported prevalence in predicting therapeutic effect according to the clinical utility analysis (48). This indicates its value in assisting clinicians in improving pretherapeutic decision-making. In addition, the predictive performance of Model 2 is superior to Model 1 and serum creatinine in all patients, respectively. This has further confirmed that the radiomics nomogram is a reliable and feasible model for predicting the treatment resistance of MPO-AAV patients with lung involvement, which is suitable to use in routine clinical practice and provides an important quantitative indicator and reference for the management of MPO-AAV patients.

This study has several limitations. First, the texture features were extracted from each MPO-AAV patient with lung involvement but not from four different kinds of lesions (alveolar hemorrhage, interstitial lung disease, pulmonary granuloma, and pleural effusion) due to them being difficult to distinguish from the sum of various lesions in fused lesions. Second, the lung involvement may be caused by other diseases rather than MPO-AAV because they were not confirmed by the percutaneous pulmonary biopsy, although patients with other lung disease such as tuberculosis and connective tissue disease-associated interstitial pneumonia were excluded. Finally, the included population was relatively small despite there being 563 MPO-AAV patients in our study. A larger sample size is necessary to further investigate the potential radiomics features to predict the treatment response of MPO-AAV patients.

In conclusion, our results indicate the feasibility of radiomics analysis in predicting treatment resistance in MPO-AAV patients with lung involvement. The radiomics nomogram constructed from a Rad-score combined with serum creatinine level is a useful, non-invasive tool for predicting the treatment resistance of MPO-AAV patients with lung involvement, which is helpful for clinicians in pretherapeutic decision-making for these MPO-AAV patients.

## Data availability statement

The original contributions presented in the study are included in the article/**Supplementary Material**. Further inquiries can be directed to the corresponding authors.

## Ethics statement

The study was approved by the Medical Ethics Committee of the Xiangya Hospital of Central South University (approval number 202108374) and the third Xiangya Hospital of Central South University for Human Studies (approval number 202109215). The patients/participants provided their written informed consent to participate in this study. Written informed consent was obtained from the individual(s), and minor(s)’ legal guardian/next of kin, for the publication of any potentially identifiable images or data included in this article.

## Author contributions

YP, WZL, and YZ conceived and designed the research. JC and TM wrote the paper. JO and PE revised the paper. JX, WGL, FL, XW, JS, HZ, and Y-OZ collected the clinical and radiological parameters of patients. JC, TM, HL, and XX analyzed data. All authors approved the final version of the manuscript.
