# Supplementary material for: Development of a radiomics nomogram to predict the treatment resistance of Chinese MPO-AAV patients with lung involvement: a two-center study
Source: Front Immunol. 2023 Jul 12;14:1084299. doi: 10.3389/fimmu.2023.1084299 (PMC10369051; doi:10.3389/fimmu.2023.1084299)
Supplement: Supplementary file 1 [file DataSheet_1.docx]

# Development of a radiomics nomogram to predict the treatment resistance of Chinese MPO-AAV patients with lung involvement: A two-center study

**Supplementary Materials and Methods**

**Appendix E1: MSCT scanning**

Enrolled patients at the two institutions had a similar MSCT scan setup. The scanning range was from the thorax inlet to the posterior costal angle for all MPO-AAV patients, who held a breath at full inspiration when undergoing the MSCT scan.

**Appendix E2: Formula of** **radiomics score:**

Radiomics score was calculated for each patient using the following formula: Radiomics score = a_1_X_1_ + a_2_X_2_ +…+ a_n_X_n_ + b (1)

Where a_n_ is the multivariate regression coefficient of the variable n, Xn is the value of the n-variable determined from the MSCT image, b is the intercept. Each selected feature was multiplied by its coefficient, and the products were added up based on the formula.

**Appendix E3: Presentation of graphical nomogram:**

The nomogram was graphically presented by proportionally converting the regression coefficients of each predictor in the model to a 0- to a 100-point scale. The effect of the variable with the highest coefficient (absolute value) is assigned 100 points. The points are added across independent variables to derive total points, which are converted to predicted probabilities.

**Supplementary Results:**

**Appendix E4:**

Rad-score in Model 1was computed based on the robust radiomics features with the following formula:

Rad-score **=** -3.94 x wavelet-LLH_firstorder_Energy + 1.9766 x wavelet-HHL _glszm_GrayLevelNonUniformity + 3.3478 x original_shapeSurfaceVolumeRatio-2.2266 x wavelet-LHL_glcm_Idmn + 1.0881 x wavelet-HLH_firstorder_Range-1.1426 x wavelet-HHH_firstorder_Median + 1.0465 x wavelet-HLH_firstorder_Skewness -1.8421 x wavelet-LLL_glszm_GrayLevelNonUniformity + 0.9 x wavelet-HLL_glcm_Idmn-0.2225

**Supplementary Table 1.** The parameters of MSCT from two institutions

| **parameters** | **Institution 1** | **Institution 2** |
| --- | --- | --- |
| CT version | SOMATOM Drive | SOMATOM Force |
| CT tube voltage | 120kV | 110kV |
| CT tube current | 90mA | 100mA |
| CT rotation time | 0.5s | 0.5s |
| CT detector collimation | 128×0.6mm | 192 × 0.6mm |
| Image matrix | 512 × 512 | 512 × 512 |
| slice thickness | 1.0mm | 1.0mm |
| slice interval | 1.0mm | 1.0mm |
| window width | 1200 | 1500 |
| window level | -600 | -600 |

**Supplementary Table 2.** The number of radiomics features extracted from each MPO-AAV patient

| Category | Original features | Wavelet features* | Total |
| --- | --- | --- | --- |
| Shape | 14 | - | 14 |
| First-order | 18 | 144 | 162 |
| Second-order |  |  |  |
| glcm | 24 | 192 | 216 |
| glrlm | 16 | 128 | 144 |
| glszm | 16 | 128 | 144 |
| ngtdm | 5 | 40 | 45 |
| gldm | 14 | 112 | 126 |
| Total | 107 | 744 | 851 |

Note: Wavelet features*=higher-order features; glcm=grey level co-occurrence matrix; glrlm=grey-level run-length matrix; glszm=grey-level size zone matrix; ngtdm=neighborhood grey tone difference matrix; gldm=grey-level dependence matrix

**Supplementary Table 3.** The screen of robust radiomics features with a multivariate logistic regression

| Variables | Coef | Std.Err | P |
| --- | --- | --- | --- |
| intercept | -0.2225 | 0.2234 | 0.3193 |
| wavelet-LLH_firstorder _Energy | -3.9400 | 0.8518 | 0.0000 |
| wavelet-HHL _glszm_GrayLevelNonUniformity | 1.9766 | 0.6150 | 0.0013 |
| original_shapeSurfaceVolumeRatio | 3.3478 | 0.7080 | 0.0000 |
| wavelet-LHL_glcm_Idmn | -2.2266 | 0.5052 | 0.0000 |
| wavelet-HLH_firstorder_Range | 1.0881 | 0.2947 | 0.0002 |
| wavelet-HHH_firstorder_Median | -1.1426 | 0.2867 | 0.0001 |
| wavelet-HLH_firstorder_Skewness | 1.0465 | 0.2851 | 0.0002 |
| wavelet-LLL_glszm_GrayLevelNonUniformity | -1.8421 | 0.6169 | 0.0028 |
| wavelet-HLL_glcm_Idmn | 0.9000 | 0.3978 | 0.0237 |

Note: Std.Err=standard error; Coef=coefficient


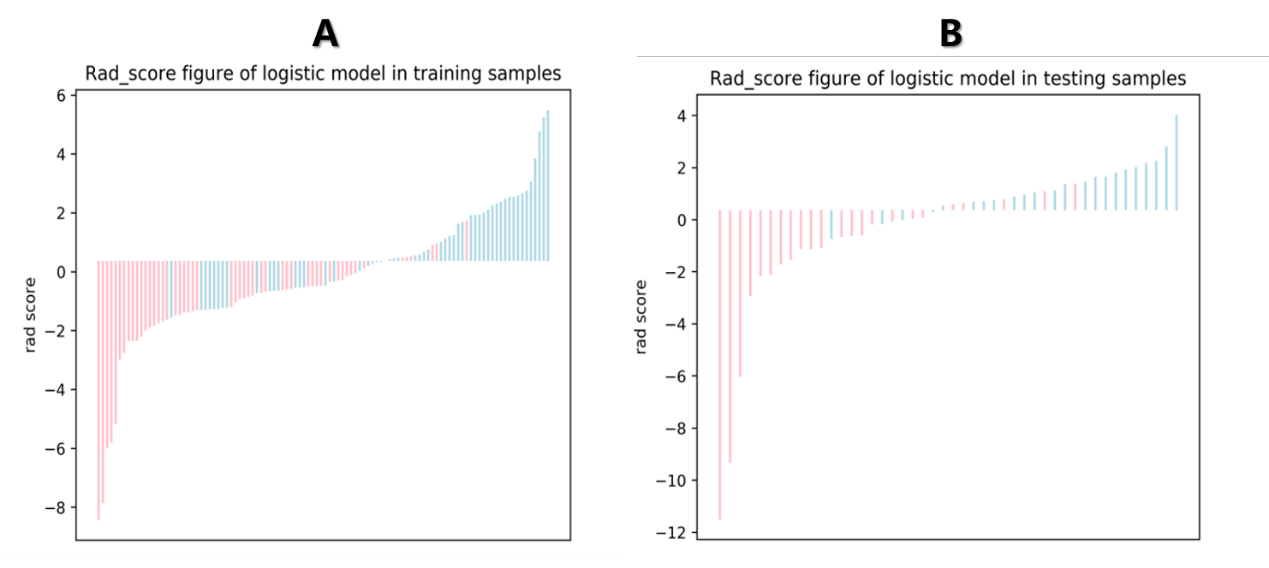


**Supplementary Fig. 1** The bar charts show the radiomics score in training (A) and test (B) cohorts. The blue bars represent scores for the treatment resistance, while the red bars represent the scores for the treatment response. The radiomics score makes a correct prediction when the blue bar is positive or the red bar is negative, and not vice versa
